# On the Diseases and Injuries of Seamen

**Published:** 1853-10

**Authors:** G. R. B. Horner

**Affiliations:** Surgeon U. S. Navy, Philadelphia


### MEDICAL EXAMINER,

AND

### RECORD OF MEDICAL SCIENCE.

NEW SERIES.—NO. CVI.—OCTOBER, 1853.

---

### ORIGINAL COMMUNICATIONS.

---

*On the Diseases and Injuries of Seamen.* By G. R. B. HORNER, M. D., Surgeon U. S. Navy, Philadelphia.

A very rare case of abscess in the left parotid gland occurred on board the Delaware in her voyage to Brazil. The patient was a seaman of 33 years, who, without any known cause, was seized with swelling and acute pain in the part, without any constitutional symptoms for four days after his admission on the sick-list. Fever then ensued, and was followed by a great increase of pain and swelling. This soon extended itself to the whole side of the face, and the lids of both eyes became tumefied so much as to intercept vision. Linseed poultices were at first applied, and upon the extension of inflammation into the surrounding parts, were mingled with a solution of super-acetate of lead. Purgative pills were taken, the parts were scarified, and suppuration having occurred on the sixth day, an incision was made into the abscess with a lancet and a large quantity of fœtid pus let out which continued to flow for many days. In the meantime the poultices were continued, and opium rubbed up with vinegar was given to relieve pain. Fever was subdued with the spiritus mindereri and tartar emetic, the blue pill was taken at night and worked off with epsom salts or magnesia and rhubarb. The abscess, however, refused to close; the neck and left angle of the lower jaw became indurated and swelled, and his constitution depraved. A bitter infusion and syrup of sarsaparilla were prescribed as needed; lead water was injected into the cavity formed beneath the ear, a lotion of sulphate of zinc was used, the black wash was also injected and a seton passed through the abscess from above downwards. An incision was likewise made beneath the left eye to let out a collection of pus formed there. He was put on a diet adapted to his condition, and at first low, then nutritious. The bowels were kept open by the purgatives mentioned, cream of tartar water, and castor oil; and tone was given to his system by the sulphate of quinine dissolved in elixir of vitriol and water, and properly sweetened. Under this treatment he was restored to health two months after his admission.

An abscess of a still more obstinate kind, bearing a strong resemblance to that of the armorer, occurred in the above ship, and was treated at the same time with the abscess just described. But the duration of the former one was much longer, that being continued a period of six months, and it was the result of a scrofulous inflammation of the left hip joint, or morbus coxarius. The subject of the affection was a sailor of small size, aged 22 years, who stated that he had fallen on the left hip six years previously, and had hurt it so much as to have been lamed for a week, but that afterwards he suffered no inconvenience until just before he was taken under treatment. Pain then came on in the left groin and its glands began to swell. Two days subsequently he had cough, general pains, and other signs of having caught cold.

The ship at this time was lying in Hampton Roads ready to sail, and only awaiting orders, and a number of her crew were affected with catarrh. As the patient seemed only affected with this, and had said nothing of his having once injured his hip joint by a fall, he was given at first only a pectoral and a dose of epsom salts and tartar emetic, and directed to use frictions to the seat of pain with vol. liniment. A soap plaster was next applied to the glands, which reduced their size, but the pain continuing, the parts beneath becoming tumid and indurated, two blisters were put on in successsion. The swelling still increasing, poultices were substituted, and excited suppuration three weeks after his admission. By this time the swelling extended throughout the groin and occupied the parts above and below Poupart's ligament. An incision was made into the swelling of the groin, a profuse discharge of bloody pus took place, and in a few days assumed a greyish hue. The nature of the disease having been previously developed satisfactorily, the patient had been prescribed the syrup of sarsaparilla twice a day in the dose of three ounces. A month afterwards another incision had to be made on the inside of the thigh, next the scrotum, to let out pus. The blue pill was given, the syrup continued, and frictions were used to relieve pain and stiffness of the thigh. He also took opiates as required, and an infusion of cinchona and gentian, after having used the syrup for more than a month. His bowels were kept open by pills and castor oil; black wash was applied to the abscess as a lotion, and when the former had closed, an issue was made over the joint with caustic potash, and beans were put into the issue formed to keep it running. Under this treatment the affected parts healed kindly though slowly; but by the twelfth of December, at the time the ship had crossed the tropics and got on the coast of Brazil, he had become pallid, emaciated and debilitated, and besides had had an ulcer formed on the sacrum from his having been bedridden two months. The left lower extremity was also soft, œdematous and swelled, the leg was flexed on the thigh, and this on the pelvis, and Amesbury's splint was applied to keep them extended, and prevent permanent flexure and shortening of the limb. To remove swelling a bandage was first applied around it from the foot upwards.

Notwithstanding all that was done for him, and his previous improvement, he became still worse on our arrival at Rio, and was seized with fever. The parts again swelled, suppuration returned upon the re-application of a poultice to the groin, and a spontaneous discharge of pus occurred at the first incision, through a fistulous opening. The fever was relieved after this, and the administration of repeated doses of tartar emetic, each consisting of the $\frac{1}{8}$ of a grain in solution. Afterwards he took the quinine mixture three times a day, and kept on the poultices as long as necessary. On introducing a probe, the abscess was found to reach several inches towards the anterior superior spinous process of the ilium. But by a continuance of the treatment adopted the patient again improved; the groin healed, and he gradually recovered the use of the limb so far as to be able to walk about the ship with a crutch. Perfect use of it was impossible, for so far as could be ascertained, the head of the femur had been entirely removed by caries, spontaneous dislocation had happened and a false joint had been formed on the dorsum of the ilium. The knee and foot were turned inwards as in accidental luxation, the limb was shortened about three inches, and the patient was barely able to touch the decks with the toes of the left foot. He was, moreover, so unlucky as to again take cold on our arrival at Montevideo, and to have the affected joint injured by concussion with a shipmate. By these mishaps his recovery was retarded, but in April he was discharged to duty, and being lame was sent home during the following summer.

All the varieties of external or tegumentary ulcers I have met with at sea, but the most numerous and difficult of cure were the varicose, indolent and venereal. The first kind were uniformly seated on one or both legs and near the enlarged veins, and the second kind were generally on the same parts, and sometimes of a scorbutic character, having been attended with puffiness and lividness of the limbs, the formation of fungus and a strong disposition to slough, which displayed itself particularly in the La Plata. The cause of this was thought to be as much owing to improper aliment as the coolness and dampness of the climate, rendered more injurious by the strong winds continually blowing up the river from the southern Atlantic; for the ship's crew were supplied abundantly with fresh provisions several times a week. In the treatment of the varicose ulcers, uniform pressure was made upon the limb with a spiral bandage and adhesive strips, numerous metallic lotions and some ointment were applied, the patient was kept at rest, the limb was elevated, and the constitution kept in or restored to a sound condition. The same rules were observed in treating indolent ulcers; the edges were kept down by lunar caustic, pressure and scarification, and they were washed generally with a solution of the sulph. of zinc, sulph. of copper or nitric acid several times a day, or sprinkled with powdered cinchona, which was often very efficient. On the coast of Brazil this was most observed, and most practised, from the general indolence there of all kinds of ulcers. Hence the cases there were also treated by the free use of internal tonics, among which the preparations of quinine were commonly preferred. Of the ointments, the citrine, red precipitate and Turner's cerate were most efficacious. As in the treatment of abscesses, the blue pill was employed, laxatives were given afterwards, and the patients dieted according to their condition. When they manifested scorbutic marks, lemonade was freely drank, and some of the bitters or tonics were administered.

In the treatment of venereal ulcers, my uniform practice was to apply the lunar caustic once or more, then to heal them up with the black wash, or a solution of sulphate of copper or zinc, and if this failed or was not effectual enough, a little mercurial ointment was rubbed near the ulcers. Sometimes the blue pill was taken, and in a few cases, when the ulcers sloughed, a solution of ʒi or ʒii of nitric acid in ℥viii. of water was applied with certain success, though very severe. If buboes followed the ulcers, they were if possible driven back by leeches or blisters, but these frequently failed, and the latter were objectionable from their inducing permanent induration of the skin as well as thickening of it. My practice, therefore, was generally, to cause suppuration by applying linseed poultices. But at Naples the virus of syphilis was so active and violent, that it quickly impregnated the system, acted like the poison of a viper on the parts, producing chancres and buboes in rapid succession, and I found that the best practice was to induce their absorption or suppuration by frictions with mercurial ointment in the course of the absorbents leading towards them. When constitutional symptoms followed they were combated with the syrup and decoction of sarsaparilla, and sometimes with blue mass in alterative doses. Other mercurial preparations were not employed at all, and salivation was never induced, yet very few instances of secondary syphilis happened, and no case was uncured, nor were any bones made carious, as under the old mercurial mode of treating syphilitic diseases, when patients were made to spit by the quart, according to John Hunter's practice. One of the most remarkable cases of secondary syphilis was that of an officer who contracted two chancres at Malta. They were healed with the nitrate of silver, black wash and lead water, without any apparent constitutional taint; no buboes formed, and yet six weeks after he was first treated for chancres, and some days after he had returned to duty, a venereal ulcer formed in both tonsils. His diathesis was highly nervous and sanguine, the ulcers were irritable and inflamed, spread extensively, were very hard to cure, and resisted the healing virtues of several gargles, as of borax, sage tea and honey, alone, and united with tincture of myrrh. When these gargles had failed, a solution of five grains of the nitrate of silver, to an ounce of water was substituted, and applied once or oftener every day with a mop. This medicine had the desired effect, and was gradually increased to 10 grains, the patient was put on low diet, took five grains of blue mass every night, and a decoction of radix sarsaparillæ ℥iv, senna ℥i, extract of liquorice the same, extract of sarsaparilla ʒi, loaf sugar ʒviii, in a gallon and a half of water, which was boiled down a third. The next day after taking the blue pill he took a dose of powdered rhubarb. Afterwards some sassafras and mezereon were added to the decoction, but proved irritating to the throat and were omitted. By the above treatment the ulcers were healed in about a month, but the fauces continued inflamed, and a scaly eruption appeared on the scalp, spread downwards over the back, and formed copper colored blotches over all the body and limbs. Not even the penis was exempted, and it became covered at the lower part with continuous scabs. This eruption was as obstinate as the ulcers in the throat, which reappeared in a short time, again healed up, and broke out again four months after they first appeared. The solution of nitrate of silver was perseveringly applied to them, and at last effected a cure with the help of constitutional remedies. In addition to those named, hot bathing in fresh and sea water, was used every day, seidlitz powders were substituted for rhubarb after the blue pill, and the skin kept perspirable by dissolving four grains of tartar emetic in a gallon of the diet drink. His condition was soon improved by this change of treatment, his throat and skin assumed their natural state and he was restored permanently to duty early in August. His recovery was a subject of joy to himself, medical attendants, and colleagues, who had been performing his duties so long, and would have probably been obliged to do so indefinitely, had he been treated according to the old routine and thoroughly mercurialized; for his irritable, sanguine temperament and partly scrofulous habit, would certainly have been aggravated by such practice, and incurable ulcerations and caries been the probable consequences. His case illustrates too the very great importance of healing chancres immediately. His were not healed, I understand, from his living on shore when they broke out, and their having been allowed to attain a large size before the medicines mentioned were applied.

Of primary syphilis many cases were treated, and most of them occurred in the Mediterranean, whose chief ports abound with every form of venereal diseases. Hence the crews of vessels allowed to go frequently on shore at them, invariably suffered much from them. That of the United States, consisting of 480 men, proved that fact, for about a fifth were infected during her cruise ending in the fall of 1838. Of these, a fourth had primary and secondary syphillis; and sixty, gonorrhoea. In the case of the latter disorder, both on board her and other vessels, as well as on land, I have found that the safest and surest plan of treatment was to combine local with constitutional treatment. This was ascertained by using each method seperately on distinct classes of the patients, learning the average time it took to cure each class, and then doing the same with regard to a third class, treated after the joint method. The average number of days for the last class was fourteen; that of the others much more. Hence the general method of treatment adopted afterwards was to confine the patients to their hammocks, open their bowels with castor oil in preference to any saline purgatives which might render the urine more irritating; and to give gum water or flaxseed tea, sometimes with a grain or two of tartar emetic to a quart, chiefly to create slight qualmishness and prevent indulgence in food. This was strictly farinaceous, and commonly fluid. Leeches were applied under the urethra, or poultices to the whole part, warm bathing was used, and injections of the superacetate of lead, from three to five grains, prescribed. This was gradually increased, now and then joined with the sulphate of zinc, or substituted by the nitrate of silver much diluted. This latter I have employed first, but have seen it produce such violent effects, as not to be able to recommend it at an early stage and in a large quantity. To relieve chordee, camphor in pills of five grs., and frictions under the urethra with mercurial ointment were frequently employed, the first article acting as an anodyne and sedative, the latter producing absorption of the coagulable lymph, preventing the expansion of the urethra from corresponding with that of other parts. Internally, we likewise gave powdered cubebs, in the doze of ʒi. two or three times a day, the oil of the same article, and balsam of copaiva in its pure state, mixed with sugar, gum arabic, and water, or made into pills with calcined magnesia, which is a very excellent method of using the copaiva, as it prevents nausea, corrects acidity, and enables the stomach to retain a much larger quantity. Hence my patients preferred the pills to any of the balsam mixtures. It mattered not whether they were combined or not with laudanum, tr. of lavender, or sweet spirits of nitre, and especially with the last, which is so ethereal as to cause the eructations to be intolerable to some persons. By the above treatment they were cured, pleasantly, and without stricture, but the former was varied when required, and if orchitis supervened, the affected part was leeched, poulticed, washed with cool astringent lotions until restored to its pristine dimensions, and when these remedies failed, the iodine ointment was successfully applied; but it occasions such excessive pains after the cuticle is stripped off by it, that few patients will submit to its reapplication.

*Cutaneous Affections.*

Besides the diseases mentioned, some of the most common met with were scabies, herpes, impetigo, acne, and urticaria. Between the tropics the miliaria occurred frequently, scarlatina north of them, and variola in every climate. Pemphigus was of rare occurrence every where; no case of measles was seen on board of any ship. Erysipelas was very rare, and the same may be stated respecting other cutaneous affections not noticed. Of scabies every grade was met with, those only affecting the fingers and arms, as well as those spreading over all parts of the body, and simulating diseases of a different kind. This was properly ascribed to neglect, want of cleanliness, and allowing patients to remain affected with itch without being reported, or from their keeping aloof from medical advice and using no remedial agents, until the skin had become thoroughly irritated by the animalculæ, and was covered with scabs. From this circumstance they may be attributed to some other cause, and are liable to be thought owing to some of the eruptive disorders. In warm climates from constant excitement kept up in the skin, this blunder is most apt to be made, particularly when the patients keep aloof and apply for no relief until forced by excessive itching. But by careful examination of the hands for the detection of the small vesicular pimples commonly found in itch between the fingers; by the fact of others having the complaint on board, and the absence of constitutional symptoms, we may satisfy ourselves of its existence in the persons being examined. In the treatment of those affected, a speedy cure was uniformly obtained by me from the use of sulphur ointment rubbed on all parts affected twice or three times a day, and the cure of some cases was hastened by giving as often from ʒi to ʒii of the flowers or lac sulphuris in molasses. A solution of the iodide of potash as a lotion is also efficacious, and is preferred by some from its not tainting the air, but I decidedly prefer the sulphur. For the hands, the most cleanly mode of applying it was to work up the flowers with soap, and to use it as the ordinary. To the body the ointment was applied twice or three times a day, and in some cases when the whole skin was infected, I generally gave the flowers in the dose of ʒi in molasses, as often. To relieve the itching immediately, and the inflammation occurring in bad cases, a solution of the super-acetate of lead was used as a lotion. Change of clothes, as much cleanliness as could be observed while using the ointment, and separation from the uninfected, were likewise enjoined, and by strict observance of this treatment the disease was uniformly soon cured and stopped from diffusion. Besides animalculæ causing scabies, I have frequently known seamen to be greatly annoyed by vermin of two kinds, the common louse and crab. For the cure of the complaint caused by the first, the razor, and a thorough washing with soap and water, prescribed by the officers of the line, are usually employed, though as much for disgrace as a remedy. But for that complaint as well as for the one occasioned by the other vermin, none can be used with greater certainty than mercurial ointment. By rubbing it in the hair, or on the parts affected, a few times, it will always act as a deadly poison to the vermin, at once kill them and remove the itching.

Herpetic eruptions were met with at long intervals, and were mostly of a mild kind, which soon healed by application of the lunar caustic, or citrine ointment; but some cases were not cured until treated constitutionally. The severest case I ever saw was that of a young muscular, plethoric seaman in the Mediterranean, who had had an attack four years before. His whole body was covered with the eruption, in widely spread or circumscribed patches, of a bright red hue, and throwing off thin scales. His scalp was affected also, and the eruption on it became pustular. He was twice on the sick list, the first time for three months, the second for four, and was each time cured with great difficulty. The remedies employed for the purpose were ol. ricini, epsom salts, sulphur and cream of tartar, to relieve the costiveness preceding the disease, the compound decoction of sarsaparilla, blue mass, minute doses of corros. sublimate in the former, the warm bath, and various lotions and ointments. Of the last named, the red precipitate and citrine were the most useful. Of the lotions, one made of corrosive sublimate, from one to two grains in an ounce of water, was by far the best, and during the several terms of treatment the cure was not effected until it had been applied. The eruption then soon began to wither; and when cured, no other relapse occurred to my knowledge. Like treatment locally was best for the ordinary ringworm, herpes zoster or shingles, acne, and impetigo. For urticaria, if induced by improper drinks or diet, as fish, sour fruits, and red wine of Minorca, emetics and cathartics were prescribed, of which epsom salts and tartar emetic to produce a double effect of purging and vomiting, or magnesia to act as an antacid and purgative, were preferred. For urticaria induced by heat of weather, or by excessive exercise, no application was comparable to aqua ammoniæ applied with a sponge to the affected parts. Though stimulating and pungent, it gives instantaneous relief, and hardly needs reapplication. It produces a like effect in other eruptions and external inflammations—erythematous, erysipelatous, and miliary; and even in gouty inflammation of the feet, I have used it with great success. At the same time that it removed this it kept up such excitement of the parts as to prevent it from becoming retrocedent. The action of ammonia is certainly very peculiar; though it stimulates, it also cools, seems to have the power of causing a disgorgement of the capillary vessels by inducing their contraction, and of producing a sedative action in them, and thus to relieve inflammation. Ammonia, moreover, by its rapid evaporation, carries off a great deal of caloric from the parts whereon it is applied, and by that means too acts remedially. But care must be taken that it be not of full strength, and is diluted with water appropriately to the case under treatment. Otherwise excoriation and unnecessary pain may be caused. When this is intense, some spirits of wine, brandy, or whiskey added to the ammonia increase its efficacy.

Scarlatina was seldom encountered, and it always happened, in the United States, during the winter, and on the eve of departure. There was no reason to believe it originated on board ship, but that it was brought from shore. In its nature it differed as it does on land. Some cases were mild, attended with slight fever and eruption; others were accompanied with high excitement at first, then by great prostration. Such was the fact with a seaman of the Brandywine at New York, who died there of scarlatina. In him it was of the most inflammatory kind, in the commencement. His pulse was very full, strong and rapid, v. s. was demanded, but soon after it his system sank, delirium came on, putrid ulceration of the throat occurred, sordes collected on the teeth, and he died on the fifth or sixth day after he was taken sick. His death, however, may be in part ascribed to his being overlooked on the second day after his admission on the list, from his having kept in an obscure place, not applying for further attention, and from the very great number of other patients to be prescribed for during the intensely cold weather we were then suffering. Most of the other cases which occurred were mild, attended with little or no fever, with a white furred tongue, having red papillæ projecting through its fur, and slight inflammation of the skin and throat, and required only laxatives, mild diaphoretics, and astringent gargles, as of alum, borax, sage tea, and tincture of myrrh, to be cured. But after the ship got to sea, another man, who had his throat very much inflamed and swelled, suddenly sank into a typhoid state and died. He had been unwell a month, was convalescing it appeared, but being confined on the berth deck with a number of persons ill of typhus fever, he evidently was infected with it, had symptoms of pneumonia, and was so much surprised when he heard that the captain's cook, in a cot near his, had just died of the former disease, that he lost all hope, repeated what he heard, "the French cook is dead," sank upon his pillow and died within an hour. He was a novice at sea, and not possessed of the fortitude and peculiar buoyancy of spirit belonging to sailors. Of all eruptive disorders, the most common, the most afflicting among them, is small pox. In all places, in all climes, they suffer from it, and of six ships of war in which I have cruised, it infected the crews of three. Three of them escaped by good luck, if not by precautionary means, but one, the United States, got it on board three times, once before she left New York in 1836, and twice during her cruise in the Mediterranean during that and the following year. The first time, only one case happened, from its being sent to the naval hospital as soon as detected. By the same precaution the Brandywine had previously got rid of the disease, at the same place, and the Delaware was equally fortunate in doing so, at Rio de Janiero. In the last instance, for want of a better place, the patient was put beneath a tarpolin tent pitched for him on a large rock, called Rat Island, opposite the city, and there treated until well. A man who had had small pox attended him, no one else than he and myself were allowed to go on the rock, and though the patient had had the primary fever and staid in the ship four days afterwards, no other person got the disease. This may be attributed to its having been modified by vaccination, of which he had a good mark on his left arm, or to the persons who were near him having been protected, or being insusceptible otherwise. He himself caught the disease merely from being on some barrels with a negro man whom he found on the island of Enxados, and conversed with for some time. The negro was convalescing, and had so little the appearance of having had small pox, that my patient did not think of danger, and allowed him to sit only a foot or two from him; nor did the latter make known these facts until two days had elapsed after he was seized with the primary symptoms, the pustules had been developed and strict enquiry was made of him regarding exposure to the complaint. Hence the cause of his not having been sooner sent out of the vessel and quarantined.

She also had a like lucky escape from contagion after her arrival in the Mediterranean. A store-ship brought a case of small pox into the harbor of Mahon, and anchored near her and held communication. The case was reported to Commodore Morris, as one of a doubtful character; I was sent to see it and found the patient as scabby as Lazarus, and could not refrain from expressing my astonishment that there should be any doubt of the nature of the case. It was reported to the Commodore, intercourse was prohibited with the store-ship, and though her commander denied the man had small pox, at my recommendation, he was sent to the Lazaretto, in charge of a man protected by vaccination, but who being not entirely so, became infected with varioloid, and had a moderate attack. This proved positively the character of the complaint and it was further proved by inquiry, and ascertaining that a case of varioloid had occurred in the above ship, a few days after she left home. In what manner she obtained pratique at Gibraltar, and again at Port Mahon is still a mystery, and it is uncertain whether it was owing to connivance on the part of the quarantine officers, their being uninformed of the cases which had occurred, or to the nature of the disease not being understood by the medical officer and others of the vessel infected. By keeping the patients in the Lazaretto until well, and then, according to my custom, by burning all things belonging to them, the squadron escaped greater contamination. Of the good effects of separating the sick from the well, and of the contrary from not doing it, proof was afforded on board the United States. While lying in the port of Alexandria a marine on guard at the star-board gangway saw a shore boat approaching with several Egyptians in it. One had marks on his face of recent small pox, the boat was hailed and ordered to keep off, but she got to the foot of the ladder before she did so, and within fifteen or more feet of the sentinel. A week after, the squadron left port he was taken sick of varioloid, got well in a few days, but infected a seaman with genuine small pox. Fortunately the ship arrived at Mahon before it attacked him, he was sent to the Lazaretto, where he staid three weeks, and had the disease in the confluent form. Yet by observing the precautions mentioned no one got the disease from him.

Of the bad effects of not excluding patients ill of it from the sound, an instance happened during the subsequent winter at Smyrna. A mulatto seaman in watering the frigate there, got small pox from some of the natives at the fountain. At the same time he was seized with a violent catarrh, or influenza, then universal in the crew, and had the other complaint so masked that he was treated for the latter several days, and until by the approach of a lamp, near his face, the eruption was discovered by me on his forehead. As soon as this occurred, he was put out of the sick bay, which was in the bow of the vessel, and hung in his cot on the outside. From there he was soon transferred after some difficulty in getting permission, to the gun deck, and near the fore hatch. Deeming that an improper place for him to remain in, as many men hung their hammocks near him, and having in vain recommended his being sent on shore, I applied for the poop cabin which was vacant, to accommodate him. The commander tried to prove that the disease was more apt to spread from there, than the gun deck, and resisted all persuasion. At last on my informing him that he must then take the responsibility, and I would send a letter to that effect, he yielded and the man was put into the cabin. It was too late, he died in a day or two, and was thrown through a port hole into the Egean sea. A sailor who had slept next to him on the gun deck, got the natural small pox from him, and before and after the ship reached Mahon, and we were able to separate him from the crew, and send him to the Lazaretto, ten others, eight men and two officers, were infected.—From a precisely similar circumstance, the small pox got on board the Macedonian on the eve of her departure from Brazil, in September, 1828. Two merchant seamen, at Rio, came to her for passage home. One was Freeman, a mulatto, and in two or three days afterwards, on his presentation at the table of Surgeon B. Ticknor, who was prescribing for the sick on the gun deck, he was found to be feverish, to have his forehead swollen and covered with variolous pimples. The surgeon forthwith got up, and reported the case to Commodore Biddle, who sent the patient without delay to the city hospital. The frigate left port, the south east trade wind soon arose, swelled her sails, and wafted her homeward; she bounded over the waves, dashing the snow white foam far from her sides. All hearts were glad, the merry sailors in the evening collected on the forecastle, and danced to the inimitable music of Miller, the sailor fiddler, and Jack Williams, a lively young negro, capered a jig to the admiration of every bystander. The next evening the same merriment was repeated, but the day after, an invalid, James Grant, who was consumptive, and had been sent from the corvette Boston to go home in the frigate, was seized with small pox. He was in no manner protected. He had neither been inoculated nor vaccinated, and most unluckily had had his cot hung in the sick bay next to the hammock of the infected mulatto. This apparently trifling incident proved a most disastrous one. Grant had the disease in the most terrible manner, the pocks on his face perfectly coalesced, became almost black, and his whole body seemed to have become decomposed, such was its fœtor. It was in vain that he was put behind a screen, abaft on the starboard side of the gun deck, and there only approached by the nurses and medical officers. The whole ship was tainted, the air even in her fore chains, outside of her, smelt of him, and every one in her liable to the contagion became affected. Inoculation was resorted to for protection as soon as the scabs could be had. On the 14th day he died, was quickly wrapped up, and lashed in his cot, with four 18lb. shot in the foot of it, was pushed over a gun, and thrown into the sea, through one of the nearest ports. No one save attendants witnessed the burial, and after all the articles used by him had been thrown after him, the deck was cleaned and sprinkled with vinegar.

After such care we hoped no other case would happen. The crew the day after had become as jolly as ever, and Miller's music was again heard. A sudden stop a second time was put to it. On the fifth day after Grant's death, Moses Nicholas, cook of the cockpit, was seized with the fatal disease, which was so violent that the eruption was suppressed, his skin became covered with numerous little pimples like those of flee bites, and he died on the seventh day, vomiting and purging blood. While he was gasping for breath, the jolly Williams, a fellow cook, who had the primary fever, got out of his hammock, sat upon a shot.box, and seeing his friend expiring, cried out, "a fair word to you Moses!"—returned soon to his hammock; in a few days he died of the fell disorder, and was likewise consigned to the deep. Five others shared the same fate, some after a short illness, others after a protracted struggle. Of the former was Samuel Parker, a marine, whose skin became purple, put forth no pustules, and lost its cuticle in large flakes. Of the latter was Alfred Curtis, a sergeant of marines, who lingered a month, and died after suffering greatly from an enormous sloughing ulcer at the lower part of his loins, which had reached a large size before he complained of it, and it was discovered. Previously he had had ulceration of the left leg, and subsequently a large abscess burst beneath the chin. Altogether forty of the crew and officers took small pox in some form, from the mishap of taking Freeman on board. Of that number, seventeen had it naturally, three had it modified by inoculation, and twenty by vaccination. Some of these persons had it very lightly; one of them would not keep from duty, nor come on the sick list, and several had merely slight fever and a few pocks which soon disappeared. Of the crew more than a hundred were inoculated as soon as the virus could be got, and only one of that number had the disease. But several afterwards had headache, pain in the loins, and immense scabs upon the parts inoculated. That on the arm of the first lieutenant, the present Commodore Salter, continued to grow until it was about the size of a half dollar, and yet he had no observable constitutional affection. The effects upon myself were simply languor and uneasiness in the loins. No one of those protected died, and those who were so by vaccination had the mildest symptoms.—Desquamation in the latter came on soonest, and, in the worst cases, was completed in a few hours. Most of the natural cases were confluent, but one of them, that of Alexander Neale, or young Turner, seaman, was remarkable for the very small number of its pustules, and their great perfection. He got well, and when with me in the Delaware, twelve years afterwards, retained no visible marks of small pox, which cannot be asserted of many who had only had varioloid. The last person who took the disease in any form was admitted on the sick list upon the 30th of September, or the fifteenth after the death of Grant. Thirty of the cases occurred within ten days from it, and within twenty from the time of the eruption being well developed upon him.—We may therefore conclude that the period of incubation was on an average ten days, though sometimes not so long, as in himself, who shewed the eruption nine days after it appeared upon Freeman, unless the former caught the contagion from the latter during the primary period before the eruption was detected.

*Treatment.*—From the oppressed condition of the patients, and the malignant type assumed by the complaint in some of them, depletion was mostly avoided; no blood was drawn from any one, the bowels were gently evacuated with castor oil or epsom salts, and calcined magnesia; an emetic was given in four of the cases, three of which were cured; cream of tartar in solution for a drink, pills of calomel, pulvis antimonialis and opium were administered frequently. Sometimes the last was left out, and spirits of nitre and camphor were used. To remove the very acute pain in the loins, suffered in most cases, laudanum in large doses was prescribed. When the sick became prostrated, port wine sangaree, and an infusion of powdered cinchona, flavored with elixir vitriol and tincture of cinchona, and having a fourth part of whiskey added to the water, were taken. The diet was farinaceous, consisting of rice and tapioca, chiefly, and cooling drinks were allowed. As much attention as possible was paid to the general comfort of the sick, their mouths were freed of the viscid saliva collected in them and the fauces; their clothes and bedding were changed,their bodies washed; lead water and dressings applied to sore parts, and the three medical officers besides the care they took of the sick during the day, divided the night into as many watches, and attended to them during it, the surgeon taking the first one, assistant surgeon Henry S. Coulter, and myself, the mid and morning. But the sick were so crowded, on the gun deck, where all the worst cases were hung in cots and hammocks, to escape the heat on the berth deck, rendered greater by the ship being between the tropics, and crossing the equator, that it was impossible they could all be made comfortable. The want of enough good bedding, the necessity of using linen almost as coarse as sail cloth, and a tempest of three days duration, from the south west, and near the line, rendered that more difficult. We were also not provided with as good a supply of medicines, provisions, and other necessaries for the sick, as we should have been, had such an epidemic been anticipated.

In this way, then, we must account for the large proportion of deaths among those affected with natural small-pox; which, including that of Freeman, who was said to have died on shore, amounted to nine, or fifty per cent. In the treatment of the cases which subsequently in various stages fell under my special care, the rule observed was to remove the most prominent symptoms as they occurred. If action was high, blood was drawn, generally, or locally, the bowels were well evacuated with epsom salts alone or combined with tartar-emetic. This was given afterwards, in the quantity of one-eighth of a grain with half an ounce of the spiritus mindereri every hour during the continuance of the primary and secondary fever, anodynes, as laudnum and sulphate of morphia in the dose of one-eighth of a grain were taken to procure sleep and relieve pain; and lemonade, cream of tartar in solution, rice and barley water, and flaxseed tea, were drank freely. Rice boiled in milk, soup, arrow-root, and other farinaceæ were allowed for diet.

In most cases a weak solution of acetate of lead in water was applied to the eyes to remove swelling and diminish the inflammation; gargles of stewed molasses and vinegar, borax, sage tea and honey, and of tincture of myrrh and alum and water, were used sometimes, and applied to the fauces to subdue irritation and remove the swelling, as well as to clear them of the accumulations of mucus and sordes. The whole body was sponged frequently with warm water, the feet bathed in hot, the pocks, especially those on the face, were early lanced and evacuated, the face was kept covered until well with a rag dipped in camphorated oil, to protect the former from air and light, the mouth was well cleansed, and the apartments of the sick were as well ventilated as necessary. From the severe catarrhal symptoms attending the cases originating at Smyrna, expectorants were given in them, and a solution of liquorice and tartar emetic was found best adapted to them.

Severe local pains were relieved by leeches, and these were frequently applied to the temples and loins, which were always much affected in the worst cases of small pox.

By the above treatment, altered according to circumstances, the seaman, Wm. Reed, who caught the disease at Smyrna, was the only one who died, and his death may properly be ascribed, in part, to the small-pox having been preceded and accompanied with the influenza, which fixed itself on the lungs and larynx, masked the more dangerous affection; and rendcred a greater loss of blood necessary than would have been drawn had he been affected with small-pox alone. He was bled three times within the first four days of his illness, had six leeches applied to the larynx, twelve to the small of the back, and sixteen to the temples, to relieve him of delirium. Altogether he lost from the arm forty-six ounces of blood, and about seventeen from leeches, reckoning that each one drew half an ounce, which is a moderate allowance for them. Notwithstanding the loss of so much blood, the inflammatory symptoms kept up almost without intermission; the face and throat swelled, the pustules formed and were confluent, on every part of his body, and he lived ten days after he was taken under treatment.

